# Mental Well‐Being and School Environment Among Czech School‐Aged Children: Health Behavior in School‐Aged Children Study

**DOI:** 10.1111/josh.70290

**Published:** 2026-11-12

**Authors:** Petra Potmesilova, Radka Zidkova, Petr Badura, Gabriela Sarnikova, Milon Potmesil

**Affiliations:** ^1^ Department of Christian Education Palacký University, St. Cyril and Methodius Faculty of Theology Olomouc Czechia; ^2^ Olomouc University Social Health Institute (OUSHI), Palacký University Olomouc Czechia; ^3^ Institute of Special Education Studies, Palacký University, Faculty of Education Olomouc Czechia

**Keywords:** adolescents, health equity, mental well‐being, school environment, school satisfaction, teacher support

## Abstract

**Background:**

This study examined associations between mental well‐being and perceptions of the school environment among Czech school‐aged children, including age and gender differences.

**Methods:**

Cross‐sectional data were obtained from the nationally representative 2022 Czech Health Behavior in School‐aged Children study. Participants were 14,588 pupils aged 11, 13, and 15 years from 246 schools. Mental well‐being was assessed using the WHO‐5 Well‐Being Index; school indicators included teacher and classmate support, school satisfaction, academic achievement, and schoolwork pressure. Associations were examined using linear and logistic regression models adjusted for gender, age, family affluence, and family support.

**Results:**

Higher mental well‐being was associated with greater teacher and classmate support, higher school satisfaction, better perceived academic achievement, and lower schoolwork pressure. Boys reported higher mental well‐being and lower schoolwork pressure than girls, while gender differences varied across the other school environment indicators. Mental well‐being, teacher support, and school satisfaction declined with age.

**Implications for School Health Policy, Practice, and Equity:**

Schools should strengthen supportive teacher and peer relationships and provide age‐ and gender‐responsive mental health support, particularly for older pupils and girls.

**Conclusions:**

Mental well‐being is closely associated with pupils' school experiences. Longitudinal research is needed to clarify directionality.

## 1 Introduction

Well‐being is a multidimensional construct encompassing various aspects of an individual's life, including positive emotions, personal development, a sense of control, meaning, and positive social relationships [1, 2]. As a subjective state, well‐being is experienced differently across individuals, social groups, and cultures [3]. According to the World Health Organization [4], well‐being reflects a state of physical, mental, and social functioning that enables individuals to cope with everyday stressors, function productively, and contribute to society [5]. In this broader perspective, well‐being is understood both as quality of life and as the capacity of individuals and societies to act with a sense of meaning and purpose, supporting equity and sustainability [4]. The WHO further distinguishes between subjective and objective dimensions of well‐being, incorporating both personal experiences and social norms.

Children's well‐being is similarly complex and plays a crucial role in their quality of life. It is closely linked to mental health, school engagement, behavior, and school attendance [6] and is influenced by a range of factors, including physical and mental health, material conditions, and social relationships [7]. Family functioning, peer relationships, health status, school performance, academic competence, and cultural context have also been identified as key determinants of children's well‐being and life satisfaction [6, 8, 9, 10, 11].

In the context of the Health Behavior in School‐aged Children (HBSC) study, well‐being is operationalized as mental well‐being and measured using the WHO‐5 Well‐Being Index [12]. This measure captures positive aspects of psychological functioning, such as feeling cheerful, calm, energetic, rested, and interested in daily life. Mental well‐being is understood as a positive state in which individuals accept themselves, function effectively in society, and realize their potential. Core components include positive relationships, self‐acceptance, culturally supported values, and realistic expectations [13]. Mental well‐being thus reflects how well individuals function psychologically and emotionally in everyday life, including their ability to cope with stress and maintain meaningful relationships [12].

Although the WHO‐5 focuses on positive affect and vitality, contemporary approaches view well‐being as a continuum from ill‐being to positive functioning rather than merely the absence of distress [14].

School satisfaction represents a closely related construct and refers to pupils' subjective evaluation of their school experiences [15]. Research consistently demonstrates associations between pupils' well‐being, life satisfaction, and the school environment, with higher well‐being linked to more positive attitudes toward school and greater engagement in learning [15]. Conversely, supportive school climates and positive school experiences contribute to higher levels of well‐being, self‐esteem, and self‐efficacy. Children who feel safe, supported, and socially integrated at school report higher well‐being than those exposed to bullying, negative relationships, or academic failure [6, 11]. School well‐being can therefore be understood as pupils' subjective cognitive evaluation of school life across multiple domains, including academic demands and social relationships [15].

Children's perceptions of well‐being and school environments have increasingly become the focus of international research. The Children's Worlds—International Survey of Children's Well‐Being (ISCWeB) [16] emphasizes children's own evaluations as a central criterion for assessing environments that support their development [17]. Findings from the HBSC study indicate that Czech children report below‐average levels of school satisfaction compared to peers in other countries [18, 19], suggesting a persistently negative relationship with school. While previous studies primarily examine how school environments influence individual well‐being [20, 21], the present study seeks to explore the reverse and less frequently researched pathway. Specifically, we examine whether children's general mental well‐being is associated with their subjective evaluation of school‐related indicators, while exploring these relationships across age groups and gender. Investigating this direction is vital, as a pupil's internal emotional state may significantly color how they perceive external academic demands, teacher behavior, and peer support.

## 2 Methods

### 2.1 Participants

The dataset included pupils from Grades 5, 7, and 9 of primary schools, corresponding approximately to ages 11, 13, and 15 years, as well as pupils attending “sekunda” and “kvarta” of multi‐year secondary schools, corresponding to Grades 7 and 9, respectively. Participants were recruited from 246 randomly selected primary and secondary schools across the Czech Republic. The response rate at the pupil level was equal to 83.1%. The sample comprises 14,588 respondents (mean age = 13.14, SD = 1.69, 50.7% boys). The respondents were divided into three age categories according to grade: 11 years—5th grade (*n* = 4334, 29.7%); 13 years—7th grade (*n* = 4767, 32.7%); 15 years—9th grade (*n* = 5487, 37.6%). For more information, see Table 1.

**TABLE 1:** Sample composition and family background characteristics by gender and age.

Group	Sample (%)	Family affluence, M (95% CI)	Family support, M (95% CI)	High family support (%)
Total	100.0	8.24 (8.20–8.27)	5.55 (5.52–5.58)	65
Boys	50.7	8.30 (8.25–8.40)	5.70 (5.69–5.77)	70
11 years	14.8	8.13 (8.03–8.22)	5.96 (5.90–6.03)	78
13 years	16.8	8.36 (8.27–8.45)	5.71 (5.65–5.77)	69
15 years	19.1	8.42 (8.33–8.51)	5.51 (5.44–5.58)	63
Girls	49.3	8.17 (8.10–8.20)	5.40 (5.32–5.41)	60
11 years	14.9	7.98 (7.89–8.07)	5.86 (5.79–5.93)	74
13 years	15.9	8.22 (8.12–8.31)	5.24 (5.17–5.31)	56
15 years	18.5	8.31 (8.22–8.40)	5.00 (4.93–5.08)	49

*Note:* Percentages in the Sample column refer to the total analytical sample (*N* = 14,588). Family affluence was assessed using the Family Affluence Scale III (FAS III; range 0–13). Family support was assessed using the Family Support subscale (FSS; range 1–7); high family support was defined as an FSS score ≥ 5.5.

Abbreviation: CI, confidence interval.

### 2.2 Instrumentation

#### 2.2.1 Mental Well‐Being

The Well‐Being Index WHO‐5, as a widely applied tool for measuring current mental well‐being (over the past 2 weeks) [22], was used to assess adolescents' well‐being and is also validated for the Czech population (https://www.corc.uk.net/). It comprises five items and a Likert response scale ranging from 0 (never) to 5 (always). The raw score is calculated as the sum of the numbers of the five responses and multiplied by 4, with perceived higher scores indicating higher well‐being. For further processing of the results, the total score was dichotomized; participants with scores 0–50 were classified as reporting lower levels of subjective well‐being (or low mood), while those with scores 51–100 were considered as reporting higher subjective well‐being. In the present Czech sample, the WHO‐5 scale demonstrated good internal consistency (Cronbach's α = 0.836).

#### 2.2.2 School Environment

The Teacher and Classmate Support Scale (TCMS) [23] is a brief two‐factor self‐report instrument of perceived social support from classmates and teachers. It consists of two subscales (Teacher Support Subscale and Classmate Support Subscale), each with three statements. The Teacher Support Scale (TSS) consists of the following items: I feel that my teachers accept me as I am; I feel that my teachers care about me as a person; I feel a lot of trust in my teachers. The Classmate Support Scale (CSS) consists of the following items: The students in my class(es) enjoy being together; most of the students in my class(es) are kind and helpful; other students accept me as I am. The respondents indicated their agreement with the statement in both subscales using a Likert scale from 0 = strongly disagree to 4 = strongly agree. The values were summed, and a score of 0–12 was created for each scale. The higher the score, the more positive the evaluation.

Three additional items assessed school satisfaction (1 = like a lot to 4 = not at all), perceived academic achievement (1 = very good to 4 = below average), and schoolwork pressure (1 = not at all to 4 = a lot). For logistic regression, responses were dichotomized as liking versus not liking school, good/very good versus average/below‐average achievement, and little/no versus some/a lot of pressure. Raw scores were retained for descriptive analyses.

In this study, both subscales showed satisfactory reliability: the Teacher Support Scale (TSS) reached a Cronbach's α of 0.812, and the Classmate Support Scale (CSS) reached a Cronbach's α of 0.774.

#### 2.2.3 Family Background

Two scales (listed below) are standardly used as control scales in HBSC studies. The HBSC Family Affluence Scale (FAS III) [24] was used to measure the family's socioeconomic status (SES). This scale is usually used in HBSC studies to assess families' material assets based on six items: number of cars, number of bathrooms, number of computers/laptops, number of holidays abroad in the past 12 months, having one's own bedroom, and dishwasher ownership. A summary score was created by summing all the FAS III‐related responses together, and the range was from 0 to 13. The scores were then used to form three basic categories that gave an overview of the family's SES. The three categories (according to empirical phenomena) were: 0–20th percentile—low SES, 21st–80th percentile—medium SES, and 81st–100th percentile—high SES. For further analysis, both the total score and the three basic categories were used.

The Family Support subscale (FSS) is a subscale that is part of the Multidimensional Scale of Perceived Social Support (hereafter MSPSS) [25]. The subscale comprises four items (Family tries to help, Get emotional help, Talk about problems, and Help make decisions) and is closely related to mental well‐being. Each item in the scale can take on a value from 1 (disagree very strongly) to 7 (agree very strongly). The total score is then calculated. In addition to the total score, we also work with a dichotomous result (low family support < 5.5 and high family support = 5.5+).

The Family Support subscale (FSS) exhibited excellent internal consistency in the current Czech sample (Cronbach's α = 0.933).

### 2.3 Procedure

Data were obtained from the nationally representative samples of Czech school‐aged children of the Health Behavior in School‐aged Children (HBSC) study. HBSC is an international collaborative research study on health and health behaviors in children. Data collection took place in the Czech Republic in May and June 2022, following the criteria set by the HBSC research protocol [26, 27]. Trained researchers supervised the completion of the online questionnaire during a one‐hour session in the schools' computer classrooms and provided standardized instructions. Participation was voluntary and anonymous. Pupils could decline to participate, discontinue participation at any time, or omit any question they did not wish to answer. Parents or legal guardians received information about the study through the school administration and could opt their children out of participation. Before completing the questionnaire, pupils confirmed their willingness to participate. The study protocol and procedures for the 2022 data collection were reviewed and approved by the Ethics Committee of the Faculty of Physical Culture, Palacký University Olomouc (reference no. 65/2020).

### 2.4 Data Analysis

The national HBSC coordinating team had cleaned the database before this secondary analysis. Item nonresponse for the study variables ranged from 0.8% to 1.5%; therefore, regression models used complete cases, with little risk of bias from missingness.

Descriptive statistics characterized the sample and variables. Gender differences were tested using independent‐samples *t*‐tests and age differences using analysis of variance. Cronbach's α assessed scale reliability.

School indicators were dependent variables. Linear regression modeled teacher and classmate support, and logistic regression modeled school satisfaction, academic achievement, and low schoolwork pressure. Predictors included WHO‐5, FAS III, and family support, with gender and age as covariates. A separate linear model examined associations of family affluence and support with mental well‐being. To align with HBSC reporting and facilitate interpretation, WHO‐5 and the three ordinal school outcomes were dichotomized using the thresholds described above; logistic results are reported as odds ratios (ORs).

To ensure that the sample was fully representative of the Czech adolescent population, post‐stratification sample weights provided by the national HBSC coordinating team were applied in all statistical analyses (both descriptive and multivariate). These ex‐post weights were calculated to align the distribution of the sample with the actual national student population (using official registry data from the Ministry of Education, Youth and Sports) based on gender, grade (5th, 7th, and 9th grades), and administrative region.

Microsoft Excel (version 2016) was used for data management. All statistical analyses were conducted using the statistical software package IBM SPSS (version 29).

## 3 Results

Tables 1 and 2 summarize the sample and descriptive findings. All four scales showed good to excellent internal consistency (α = 0.774–0.933).

**TABLE 2:** Mental well‐being and school environment indicators by gender and age.

Group	WHO‐5, M (95% CI)	Higher well‐being (%)	Teacher support, M (95% CI)	Classmate support, M (95% CI)	School satisfaction (%)	Good academic achievement (%)	Low schoolwork pressure (%)
Total	55.30 (54.90–55.70)	60	7.22 (7.17–7.26)	7.13 (7.08–7.17)	61	57	55
Boys	60.70 (60.15–61.15)	71	7.30 (7.27–7.40)	7.37 (7.31–7.44)	61	56	62
11 years	64.61 (63.67–65.55)	77	8.13 (8.02–8.23)	7.53 (7.43–7.63)	69	62	71
13 years	59.30 (58.44–60.17)	69	7.12 (7.01–7.23)	7.18 (7.07–7.29)	59	56	56
15 years	58.22 (57.40–59.03)	68	6.78 (6.66–6.90)	7.43 (7.31–7.54)	54	52	61
Girls	49.90 (49.37–50.41)	49	7.09 (7.02–7.15)	6.88 (6.82–6.94)	63	59	48
11 years	56.92 (56.00–57.84)	62	8.26 (8.16–8.36)	7.16 (7.06–7.26)	75	62	66
13 years	47.50 (46.62–48.38)	45	6.62 (6.52–6.73)	6.66 (6.55–6.76)	59	57	38
15 years	45.34 (44.51–46.17)	41	6.36 (6.25–6.47)	6.81 (6.70–6.93)	55	58	39

*Note:* WHO‐5 scores range from 0 to 100, with higher scores indicating better mental well‐being. Teacher Support Scale and Classmate Support Scale scores range from 0 to 12, with higher scores indicating greater perceived support. School satisfaction represents pupils reporting that they liked school “a lot” or “a bit.” Good academic achievement represents “very good” or “good” perceived achievement. Low schoolwork pressure represents “not at all” or “a little” pressure. Percentages are based on valid responses for the respective variables.

Abbreviation: CI, confidence interval.

### 3.1 Mental Well‐Being

Approximately 60% of Czech pupils were classified as having higher mental well‐being. As shown in Table 2, boys reported significantly higher mental well‐being than girls, *t*(13,755) = 29.35, *p* < 0.001. Mental well‐being also differed significantly across age groups, decreasing with age in both genders, *F*(2, 13,753) = 199.96, *p* < 0.001. The decline was more pronounced among girls, whose mean WHO‐5 score decreased from 56.92 at age 11 to 45.34 at age 15; among boys, the corresponding means were 64.61 and 58.22.

### 3.2 School Environment

Descriptive results for the school environment indicators are presented in Table 2. Perceived teacher support declined with age in both genders. Overall, boys reported slightly higher teacher support than girls, although 11‐year‐old girls reported marginally higher support than boys of the same age. Classmate support showed a less consistent age pattern. Boys generally reported higher classmate support than girls, but neither gender showed a continuous decline across all three age groups.

Overall, 61% of respondents reported liking school “a lot” or “a bit,” 57% rated their academic achievement as good or very good, and 55% reported little or no schoolwork pressure. School satisfaction declined with age in both genders. Gender differences were particularly apparent in schoolwork pressure: 62% of boys, compared with 48% of girls, reported little or no pressure. Among 13‐ and 15‐year‐old girls, these proportions were 38% and 39%, respectively.

### 3.3 Family Background and Mental Well‐Being

Linear regression analysis indicated that family affluence and family support were both significantly and positively associated with mental well‐being. Together, the Family Affluence Scale and Family Support subscale explained 18% of the variance in mental well‐being, *F*(2, 12,966) = 1437.05, *p* < 0.001. Family support showed a stronger association with mental well‐being, *t* = 52.67, *p* < 0.001 than family affluence, *t* = 6.47, *p* < 0.001.

### 3.4 Mental Well‐Being and School Environment by Age and Gender

Higher mental well‐being was associated with all five school indicators (all *p* < 0.001; Table 3). Descriptively, boys reported greater teacher and classmate support and lower pressure. After adjustment, girls had higher teacher support, school satisfaction, and perceived achievement but lower odds of little or no pressure; gender was unrelated to classmate support.

**TABLE 3:** Regression coefficients and odds ratios with 95% confidence intervals for associations between mental well‐being, sociodemographic factors, and school‐related indicators.

	Dependent variables				
	Linear regression (*B*)		Logistic regression (OR)		
Independent variables	Classmate support (0–12)	Teacher support (0–12)	School satisfaction	Academic achievement	Low schoolwork pressure
Gender (girls vs. boys)	−0.078 (−0.167–0.011)	**0.181 (0.093–0.269)** ***	**1.475 (1.362–1.598)** ***	**1.328 (1.232–1.432)** ***	**0.646 (0.599–0.698)** ***
Age 13 vs. 11 years	−0.080 (−0.187–0.028)	**−0.925 (−1.031–−0.820)** ***	**0.652 (0.592–0.719)** ***	**0.910 (0.831–0.997)** *	**0.424 (0.385–0.466)** ***
Age 15 vs. 11 years	**0.230 (0.120–0.339)** ***	**−1.116 (−1.224–−1.008)** ***	**0.569 (0.516–0.628)** ***	**0.840 (0.766–0.921)** ***	**0.518 (0.470–0.570)** ***
WHO‐5	**0.034 (0.031–0.036)** ***	**0.032 (0.030–0.034)** ***	**2.996 (2.757–3.257)** ***	**1.833 (1.692–1.986)** ***	**2.692 (2.481–2.920)** ***
FAS III	0.001 (−0.018–0.020)	**−0.067 (−0.086–−0.048)** ***	0.947 (0.890–1.007)	**1.247 (1.176–1.323)** ***	0.986 (0.928–1.048)
FSS	**0.227 (0.199–0.256)** ***	**0.373 (0.345–0.400)** ***	**1.780 (1.639–1.934)** ***	**1.591 (1.468–1.724)** ***	**1.564 (1.439–1.699)** ***

*Note:* WHO‐5—Well‐Being Index Scale (0–100) for Classmate Support and Teacher Support; WHO‐5—Well‐Being Index Scale (dichotomy) for School satisfaction, Academic achievement, Low schoolwork pressure; FAS III—Family Affluence Scale (0–13) for Classmate Support and Teacher Support; FAS—Family Affluence Scale categorical (20/60/20) for School satisfaction, Academic achievement, Schoolwork pressure; FSS—Family Support subscale (1–7) for Classmate Support and Teacher Support; FSS—Family Support subscale dichotomy (5.5) for School satisfaction, Academic achievement, Schoolwork pressure; Age group was entered as a categorical variable, with the youngest group (Grade 5, corresponding approximately to age 11) serving as the reference category. B denotes an unstandardized linear regression coefficient; OR denotes an odds ratio.

*** *p* < 0.001.

* *p* < 0.05.

## 4 Discussion

This study examined the association between mental well‐being and school‐related indicators among Czech children. Our findings support the hypothesis that higher subjective well‐being is consistently linked with more positive perceptions of the school environment. While we modeled well‐being as the independent variable to shed light on how internal psychological health relates to subjective school experiences, we recognize that this dynamic is likely reciprocal. A positive school environment promotes well‐being, and in turn, a mentally healthy child is likely to experience and evaluate school more favorably. The findings should be interpreted within the specific cultural and educational context of the Czech Republic. The Czech school system continues to reflect traditional social norms, including gender stereotypes related to pupils' behavior and abilities [28, 29]. Although inclusive education policies emphasizing competencies over knowledge are currently promoted, their implementation remains challenging and is accompanied by the relatively low social prestige of the teaching profession [30, 31, 32, 33]. In addition, strong performance pressure associated with entrance examinations to secondary schools persists and contributes to school‐related stress [34].

The results revealed a statistically significant relationship between perceived mental well‐being and satisfaction with the school environment. Pupils with higher mental well‐being tended to report more positive school experiences. Age and gender were also significantly associated with perceptions of the school environment, with older pupils reporting lower trust in teachers and lower school satisfaction. Girls generally reported lower well‐being and higher levels of school‐related stress, which is consistent with previous research [19]. Satisfaction with the school environment decreased with increasing age for both genders, a trend also observed in other studies [35, 36, 37].

In line with previous international reports from the HBSC study, the literature suggests that Czech pupils consistently report lower levels of mental well‐being and school satisfaction than their peers in many other participating countries. Our findings, although focused solely on the Czech cohort, reinforce this concern [19, 38]. These findings suggest that Czech children have a persistently weaker relationship with school. Although the causes of this phenomenon were not examined in detail, previous research points to strong academic orientation, traditional teaching methods, and high parental expectations as potential contributing factors [39, 40, 41, 42].

Consistent with previous research, higher mental well‐being was associated with more favorable perceptions across several dimensions of school experience, including teacher and classmate support, school satisfaction, perceived academic achievement, and schoolwork pressure [35, 43, 44, 45, 46, 47, 48, 49, 50, 51, 52]. The present study extends this evidence by demonstrating that these associations are observable across multiple dimensions of school experience in a large, nationally representative sample of Czech pupils and remain significant after accounting for age, gender, family affluence, and family support. However, mental well‐being and perceptions of school are also likely to be shaped by factors not examined in the present study, including emotional regulation, physical and mental health, personality characteristics, previous school experiences, and relationships outside school [35, 43, 44, 45, 46, 47, 48, 49, 50, 51, 52, 53]. The findings should therefore not be interpreted as indicating that the school environment alone determines pupils' mental well‐being.

The concurrent associations of higher mental well‐being with greater perceived teacher support, higher school satisfaction, and lower schoolwork pressure are consistent with previous evidence concerning the importance of supportive school relationships and manageable school demands [54]. These findings may reflect mutually reinforcing processes: pupils with higher mental well‐being may perceive relationships and school demands more positively, while supportive interactions with teachers and a less stressful school experience may, in turn, contribute to better mental well‐being. The cross‐sectional design does not allow these pathways to be distinguished. Nevertheless, the consistency of the associations across relational and academic dimensions suggests that mental well‐being should not be viewed solely as an individual characteristic but also in relation to the social and organizational conditions in which learning takes place.

Schoolwork pressure was a relevant feature of pupils' school experience, with approximately 45% of respondents reporting some or a lot of pressure. Previous research indicates that high academic demands and frequent assessment may increase stress and anxiety and negatively affect well‐being and academic self‐esteem [55, 56]. Supportive relationships with teachers and peers may help pupils manage these demands and buffer some of their adverse effects [57, 58]. Although the present study did not directly test such buffering mechanisms, the concurrent associations of mental well‐being with teacher support, classmate support, and schoolwork pressure reinforce the importance of considering both the relational and academic dimensions of the school environment. Teacher–pupil interactions, opportunities to experience mastery, and supportive educational practices may therefore represent important components of a school environment conducive to pupils' well‐being [59, 60, 61].

The age and gender patterns provide further context for these associations. The decline in mental well‐being and perceived teacher support with age was particularly pronounced among girls, who also reported considerably greater schoolwork pressure than boys at ages 13 and 15. These findings are consistent with previous evidence of age and gender differences in adolescent well‐being and school experience [35, 36, 37, 56, 62, 63, 64, 65, 66]. They also suggest that a uniform approach to school mental health may not respond equally well to the needs of all pupils. However, because the study did not examine the mechanisms underlying these differences, possible explanations—including developmental changes, gendered expectations, coping processes, academic demands, and differences in help‐seeking—should be investigated in future longitudinal research.

### 4.1 Implications for School Health Policy, Practice, and Equity

The findings support a whole‐school approach in which mental health promotion is integrated into everyday teaching, relationships, and school organization rather than being limited to counseling for pupils already experiencing difficulties. Teacher availability, respectful communication, opportunities for pupils to seek help, supportive peer relationships, and the management of academic demands should be considered components of school health promotion.

At the practice level, schools may benefit from routinely monitoring pupils' mental well‐being, perceived teacher and classmate support, school satisfaction, and schoolwork pressure. Such monitoring could help identify unfavorable developmental trends before they become established problems. Professional development should also equip teachers to recognize changes in pupils' well‐being, respond supportively, and refer pupils for additional assistance when needed. School counselors and other support professionals should be integrated into this process rather than functioning only as services used after difficulties have escalated.

From an equity perspective, the less favorable outcomes among older pupils and girls indicate that a uniform approach may not meet the needs of all pupils equally. Universal mental health promotion should therefore be combined with proportionate and age‐ and gender‐responsive support. Schools should examine whether their assessment practices, expectations, access to supportive adults, and opportunities for participation create unequal burdens or barriers for particular groups. Because the present study did not test specific interventions or causal mechanisms, these recommendations should be evaluated through longitudinal and school‐based intervention research.

### 4.2 Limitations

The cross‐sectional design precludes causal or directional conclusions: well‐being may shape school perceptions, while supportive experiences may promote well‐being. Longitudinal and intervention studies are needed to test these pathways.

All measures were self‐reported at one time point and may reflect shared method variance, current mood, response tendencies, or social desirability. Nevertheless, pupils' perceptions are central to their school experience.

Dichotomization facilitated interpretation and followed HBSC practice but reduced variability within response categories. The study also lacked school‐level identifiers and could not assess school climate, assessment practices, counseling provision, school size, or between‐school differences.

The nationally representative sample supports generalization within Czechia, but findings may not transfer directly to education systems with different structures, assessment, or approaches to school mental health.

## 5 Conclusions

Among Czech pupils aged 11–15, higher mental well‐being was associated with greater school satisfaction and more favorable perceptions of school. Girls reported lower well‐being and greater pressure than boys, and well‐being declined with age, consistent with HBSC findings [67]. Adolescence is therefore an important period for school‐based mental health promotion.

School health interventions should combine supportive environments with age‐ and gender‐responsive mental health support to improve pupils' school experiences and well‐being.

## Funding

This work was supported by the Univerzita Palackého v Olomouci (IGA CMTF_2024_001) and the Ministerstvo Školství, Mládeže a Tělovýchovy (CZ.02.01.01/00/22_008/0004583). This work was made in the framework of the project ReDiKid: Resilient Kid in Digital World, reg. No. CZ.02.01.01/00/23_025/0008686 co‐funded by the EU.

## Ethics Statement

The study protocol and procedures for the 2022 data collection were reviewed and approved by the Ethics Committee of the Faculty of Physical Culture, Palacký University Olomouc (reference no. 65/2020). Parents or legal guardians were informed about the study and could opt their children out of participation. Before completing the questionnaire, pupils confirmed their willingness to participate. Participation was voluntary and anonymous.

## Conflicts of Interest

The authors declare no conflicts of interest.

## Data Availability

The data that support the findings of this study are available from the corresponding author upon reasonable request.
